# Patient‐ and family‐reported satisfaction in child and adolescent psychiatric inpatient care: An exploratory descriptive survey

**DOI:** 10.1002/pcn5.70445

**Published:** 2026-11-15

**Authors:** Keita Yamamoto, Masahide Usami

**Affiliations:** ^1^ Department of Social Work, National Kohnodai Medical Center Japan Institute for Health Security Ichikawa Japan; ^2^ Department of Child and Adolescent Psychiatry, National Kohnodai Medical Center Japan Institute for Health Security Ichikawa Japan

**Keywords:** adolescent psychiatry, inpatients, mental health services, patient satisfaction, surveys and questionnaires

## Abstract

**Aim:**

To describe and compare patient‐ and family‐reported satisfaction with child and adolescent psychiatric inpatient care in a Japanese inpatient unit.

**Methods:**

Patients aged 8–15 years discharged from a single Japanese inpatient unit and their families completed an anonymous web questionnaire with separate patient and family versions. Overall satisfaction was rated on a 10‐point scale, and respondents who reported interaction rated the usefulness of five professional groups. Because responses were unlinked, 10 probable patient–family pairs identified from submission times were used in sensitivity analyses.

**Results:**

Invitations were handed to 61 patients and their families at discharge; 52 family members (85.2%) and 26 patients (42.6%) responded. Family members reported higher satisfaction than patients (mean 8.14 [SD 1.83] vs. 6.27 [SD 2.15]; medians 8 vs. 7; mean difference 1.87, 95% confidence interval [CI] 0.87–2.86), and the lower 95% CI limit was at least 0.73 in all sensitivity analyses addressing dependence between the groups. The maximum score was given by 25.5% of family members and 3.8% of patients. Among respondents who rated each professional group, a higher proportion of family members than patients rated child and adolescent psychiatrists (98.1% vs. 69.2%) and nurses (92.3% vs. 73.1%) as useful, whereas proportions for psychiatric social workers, clinical psychologists, and teachers were similar or higher among patients. These profession‐specific comparisons were descriptive only.

**Conclusion:**

Patients rated inpatient care less favorably than family members. Family reports should complement, not replace, patients' own evaluations, and future multicenter studies should use matched patient–family dyads.

## INTRODUCTION

Child and adolescent psychiatric inpatient treatment is a critical component of care for severe mental health crises, including acute psychosis, suicidal ideation, self‐harm, severe eating disorders, and other conditions requiring intensive, multidisciplinary care.^1^, ^2^, ^3^, ^4^ Although inpatient treatment provides stabilization and safety, its quality should not be evaluated solely by symptom reduction, risk containment, or family reassurance. Patient‐reported experience and satisfaction are increasingly regarded as core dimensions of quality because they reflect whether patients feel listened to, understood, respected, adequately informed, and safe within the therapeutic environment.^5^, ^6^, ^7^

This perspective is important in child and adolescent psychiatry, where treatment engagement depends not only on clinical need but also on perceived autonomy, trust in professionals, privacy, and acceptability of daily care.^8^ Adolescents' ratings of inpatient care have been related to treatment outcomes more often than parents' ratings,^9^ although satisfaction explains only limited additional variance in clinical outcomes,^10^ and predictors of satisfaction differ between young people and parents.^11^, ^12^

Family perspectives remain essential because parents and caregivers are key partners in treatment planning, discharge preparation, and ongoing support.^13^ However, family satisfaction cannot be assumed to represent the patient's own experience.^14^, ^15^ Parents often evaluate care through safety, crisis management, physician communication, and caregiving burden, whereas patients directly experience hospitalization, including ward rules, restrictions, peer relationships, autonomy, privacy, and daily interactions with staff.^8^, ^9^, ^16^ Comparing patient and family evaluations can therefore show how the two groups experience inpatient care differently.

Lower patient satisfaction should not be dismissed as immaturity, oppositionality, or an inevitable consequence of hospitalization but instead regarded as a potential indicator of unmet needs in communication, shared decision‐making, privacy, or the therapeutic relationship.^5^, ^6^, ^16^

In Japan, evidence regarding patient and family satisfaction with child and adolescent psychiatric inpatient treatment remains limited.^17^, ^18^, ^19^ Validated instruments such as the German BEST questionnaires^20^ and the UK Experience of Service Questionnaire (ESQ)^21^ were developed in Western healthcare systems and do not map directly onto the multidisciplinary staffing structures (e.g., high reliance on psychiatric social workers [PSWs] and hospital‐class teachers) or the statutory admission framework in Japan.^22^ Measures used in routine inpatient care are also often not developmentally adapted for young people.^16^

Previous inpatient studies have mainly used validated multidimensional instruments and parallel adolescent and parent versions. The BEST questionnaires were evaluated in 1582 adolescents and 1998 parents across seven German inpatient units; parents generally reported higher satisfaction than adolescents, and agreement in 815 paired responses was moderate.^20^ Another inpatient study found that adolescents were less satisfied than their parents and that adolescents' ratings, unlike parents' ratings, were frequently related to treatment outcomes,^9^ and a study in child and adolescent mental health services examined whether predictors of satisfaction differ between adolescents and parents.^12^ To our knowledge, patient‐ and family‐reported satisfaction with child and adolescent psychiatric inpatient care has not been described in Japan, where multidisciplinary teams include PSWs and hospital‐class teachers and admissions are governed by the Mental Health and Welfare Act.^22^ The present study adds a description of how patients and families in one Japanese unit rated overall care and each professional group.

Accordingly, this single‐center exploratory study aimed to (i) descriptively compare overall treatment satisfaction between child and adolescent psychiatric inpatients and family respondents using unlinked samples, (ii) describe evaluations of different multidisciplinary professionals among respondents who reported interaction with them, (iii) explore clinical and treatment‐related factors associated with satisfaction, including admission type and behavioral restrictions, and (iv) inform the design of future prospective multicenter studies in Japan.

## METHODS

### Study design and setting

This cross‐sectional survey used an anonymous, self‐administered online questionnaire to evaluate patient‐ and family‐reported perspectives on child and adolescent psychiatric inpatient treatment. The survey was conducted as part of the Ministry of Health, Labour and Welfare Research Grant project, “Research on Promoting Multidisciplinary Collaboration in Child and Adolescent Psychiatric Care” (Grant No. 23G1013). It was coordinated by the Department of Child and Adolescent Psychiatry at National Kohnodai Medical Center, Japan Institute for Health Security, a national tertiary psychiatric facility specializing in child and adolescent mental health.

The target population comprised children and adolescents discharged from psychiatric inpatient units and their parents or guardians at institutions participating in the Japanese Council of Child and Adolescent Psychiatric Institutions. The survey was part of a nationwide survey conducted across these institutions; this report analyzes only the responses from our institution (National Kohnodai Medical Center). The same form was used at all participating institutions, and respondents selected their hospital in the form; all 78 responses analyzed here identified National Kohnodai Medical Center. Between May 2024 and March 2026, invitation flyers were handed to 61 patients and their families at discharge at our institution, and reminders were sent afterward; responses submitted by May 31, 2026 (data cutoff) were included. These 61 patients were all patients aged 8–15 years discharged from the unit during this period, excluding two children living in child welfare facilities, who were not eligible; flyers were handed to all eligible patients and their families. Given the exploratory nature of this survey, formal sample size calculations a priori were not performed; rather, all eligible patients discharged during the survey period were invited consecutively, and responses received by the pre‐planned data cutoff were analyzed. The survey, administered using Microsoft Forms, employed questionnaires developed by an expert panel of child and adolescent psychiatrists, nurses, psychologists, and PSWs to ensure face validity for the Japanese child and adolescent psychiatric service context. Participants reviewed a study information statement and proceeded only after providing consent. The statement described the study purpose, period, eligibility, survey items, anonymization procedures, access to the study protocol, handling of personal information, contact information, and that participation was voluntary.

The approved study period extended from ethics committee approval through March 31, 2027. The questionnaire collected respondent type, patient demographics, hospitalization characteristics, diagnosis, admission status, treatment components, multidisciplinary professional involvement, overall evaluation of inpatient treatment, and usefulness ratings for treatment components and professional interactions. Responses were anonymized at submission and analyzed without individual identifiers.

All responses analyzed were from the 45‐bed child and adolescent psychiatric inpatient unit at National Kohnodai Medical Center, which had a mean length of stay of 223.2 days in fiscal year 2025. The ward multidisciplinary team comprises 6 full‐time child psychiatrists, 32 nurses, 3 clinical psychologists, 2 PSWs, and 3 certified hospital‐class teachers. The study followed the Strengthening the Reporting of Observational Studies in Epidemiology (STROBE) and Consensus‐Based Checklist for Reporting of Survey Studies (CROSS)^23^ guidelines. Completed checklists are provided in Supporting Information S1: Supplementary Materials.

### Participants and survey administration

Eligible participants were children and adolescents aged 8–15 years discharged from psychiatric inpatient treatment and their parents or guardians. Children living in child welfare facilities were not eligible because the family questionnaire would have been answered by facility staff rather than by a family member (two such children were discharged during the survey period). Patient and family versions of the questionnaire were developed within a single online form.

At discharge, administrative staff provided patients and families with invitation flyers containing the web link and a QR code for the form. Respondents first indicated whether they were the patient or a family member, and the form then displayed the corresponding version. Participants completed the web‐based questionnaire independently at home; because reminders were sent after discharge, the interval between discharge and response varied. Because responses were anonymous and the form did not restrict the number of responses per device, duplicate submissions could not be fully excluded. Patient and family responses contained no linkage identifiers. Because a patient and a family member could both respond about the same admission, the two groups are not statistically independent (see Statistical Analysis).

The analytic sample comprised child and adolescent psychiatric patients and family respondents, primarily mothers and fathers. A total of 78 respondents completed the questionnaire and were included in the final analysis, comprising 52 family respondents and 26 patient respondents.

### Measures

The patient and family versions were developed from the study information materials, ethics committee‐approved survey content, and expert clinical review to ensure face validity. The questionnaire was designed as a service‐evaluation instrument for this exploratory study rather than a validated psychometric scale such as the ESQ.^21^ The questionnaire items were generated based on routine clinical feedback and existing ward evaluation formats. While formal cognitive pretesting with children and adolescents or their parents was not conducted prior to implementation, the expert panel deemed the visual simplicity of the 10‐point scale appropriate for school‐aged children (≥8 years). As noted, this ad hoc instrument has not been psychometrically validated. Rather than adding Japan‐specific items to a validated instrument, we developed a questionnaire tailored to current child and adolescent psychiatric inpatient treatment in Japan (e.g., PSWs, hospital‐class teachers, medical protective admission, and the treatment components provided in Japanese units), so that all items referred to services that patients and families had actually encountered. This choice limits comparability with previous studies, and the measurement properties of the questionnaire are unknown. Supporting Information S1 reproduces both questionnaires as they were displayed to respondents in Microsoft Forms (screen captures of the Japanese form), with an English translation of every item and response option and a table showing how each response was coded for analysis.

Both versions collected information on respondent type, treatment components received, multidisciplinary professional involvement, overall evaluation of inpatient treatment, and usefulness ratings for treatment components and professional interactions. Child‐level background items were obtained from patient respondents only (see below).

#### Overall satisfaction

Overall evaluation of inpatient treatment was measured using a single 10‐point item ranging from 1 (“not good at all”) to 10 (“very good”). Patients evaluated their own inpatient treatment, whereas parents or guardians evaluated the child's treatment from the family perspective. One respondent who identified as the patient answered the patient background items and then, because of a branching error in the online form, the evaluation items of the family version; these answers were analyzed as that patient's responses (Supporting Information S1, Part B).

#### Respondent and patient background

Respondents selected one of four respondent types: patient (the child who had been admitted), family member (mother), family member (father), or family member (grandparent). The patient questionnaire collected the patient's sex, age, length of hospitalization, reasons for admission, diagnosis, diagnosis awareness, admission status, and changes in admission status during hospitalization. Corresponding child‐level items were included in the family questionnaire but, because of the branching settings of the online form, were not displayed to family respondents; therefore, no family‐reported child characteristics were obtained.

#### Reasons for admission and diagnoses

Reasons for admission included school non‐attendance, eating refusal or inability to eat, violent behavior at home, inability to stop gaming, temper outbursts, physical health problems, self‐harm or suicidal thoughts, depressed mood, uncontrollable anxiety, physician recommendation, and other. Diagnoses included attention‐deficit/hyperactivity disorder, autism spectrum disorder, anorexia nervosa or other eating disorders, depression, anxiety disorder, obsessive‐compulsive disorder, tic disorder, conduct disorder or oppositional defiant disorder, gaming disorder, school refusal or social withdrawal, and other.

#### Treatment components received

Treatment components were presented in two grids: interviews with the attending physician, psychologists, PSWs, nurses, and occupational therapists, and seclusion or restraint (Part 1); and group therapy or activities, pharmacotherapy, ward recreation, occupational therapy, cognitive behavioral therapy, in‐hospital classes, seclusion or restraint, and discharge support meetings (Part 2). For each row, respondents selected one of three options (received, not received, or don't know); multiple selections were not possible within a row, and rows left blank were coded as not answered. Seclusion or restraint appeared in both grids, and the first appearance was analyzed. In the patient form, the cognitive behavioral therapy row of Part 2 was displayed with the label “occupational therapy,” so that occupational therapy appeared twice and respondents to the patient form did not see a cognitive behavioral therapy option in this item. Patient responses to this row were therefore not analyzed; the family form displayed the correct label.

#### Professional involvement and evaluations

For physicians, nurses, psychologists, PSWs, occupational therapists, childcare workers, and teachers, respondents indicated whether the professional had been assigned to the patient (assigned, not assigned, or don't know); this item asked about assignment, not about contact. In a separate grid, respondents rated the usefulness of their relationship with each professional group (attending physician, nurses, psychologists, PSWs, occupational therapists, childcare workers, and teachers) on a five‐point scale (very useful, useful, neither, not very useful, and not useful at all); every row also offered the option “had no opportunity to interact.” Analyses focused on child and adolescent psychiatrists (the attending physician, all of whom were child and adolescent psychiatrists in this unit), nurses, clinical psychologists, PSWs, and teachers.

#### Usefulness ratings of treatment components

Respondents rated interviews, pharmacotherapy, group therapy or activities, ward recreation, occupational therapy, cognitive behavioral therapy, in‐hospital classes, and seclusion or restraint using the same five‐point ordinal scale, ranging from “not useful at all” to “very useful,” with the same additional option “had no opportunity to interact” (shown as “no opportunity” in Supporting Information S2: Table S2).

#### Clinical characteristics

Admission status was categorized as voluntary admission, medical protective admission, or unknown. Changes in admission status during hospitalization were assessed as one change from medical protective admission to voluntary admission, one change from voluntary admission to medical protective admission, multiple changes, or unknown. Experience of behavioral restriction (seclusion or restraint) was assessed in the clinical context of the Japanese Mental Health and Welfare Act.^22^ Length of hospitalization, patient age, and sex were also summarized.

### Statistical analysis

All variables were summarized using descriptive statistics. Continuous and ordinal variables were summarized using means, standard deviations, medians, and ranges, as appropriate, whereas categorical variables were summarized as counts and percentages. Because item‐level missing data were present, available‐case analyses were performed, and denominators were specified for each analysis.

Responses were anonymous and could not be linked by identifiers. However, submission times showed that 10 patient responses were submitted within 3 minutes of a family response; we regarded these as probable pairs from the same admission (in one case, two patient responses and two family responses were submitted consecutively, and pairs were assigned in submission order; the alternative assignment gave identical results). The primary comparison of overall satisfaction was therefore presented descriptively as full score distributions, medians, and means, with the mean difference and its 95% confidence interval (CI; Welch), together with the Hodges–Lehmann estimate of the median difference. To address the dependence between groups, we conducted three sensitivity analyses: (i) the partially overlapping samples *t*‐test (Tnew2), which treats the probable pairs as paired and the remaining responses as independent observations^24^; (ii) exclusion of the family responses of probable pairs; and (iii) exclusion of the patient responses of probable pairs. In addition, if each invited admission contributed at most one response of each type, at least 17 admissions (26 + 52 − 61) were represented in both groups; we therefore recalculated the 95% CI of the mean difference assuming 17–26 pairs with within‐pair correlations from −0.5 to 0.5. Excluding the patient who answered the family version of the evaluation items gave a mean difference of 1.94 (95% CI, 0.92–2.95; 51 vs. 25 respondents). Because further unidentified pairs may exist, the Mann–Whitney *U* test, which assumes independent samples, is reported as supplementary information only, with the effect size *r* = *Z*/√*N*. Within the probable pairs, the numbers of pairs in which the family score was higher than, equal to, or lower than the patient score were counted.

For professional evaluations, the denominator for each professional group comprised respondents who rated the usefulness of their interaction with that group; respondents who selected “no opportunity to interact” or left the item blank were excluded. This self‐reported interaction criterion was applied identically to all professional groups and to both respondent groups. The assignment item is reported descriptively and was not used to define denominators. Positive evaluation was defined as “useful” or “very useful.” The dependence between patient and family responses described above also applies to these comparisons, and the unlinked data did not allow it to be modeled. Profession‐specific results are therefore presented descriptively as full response distributions and proportions, without CIs or tests that assume independent samples. Distributions of treatment components received and of their usefulness ratings are presented in Supporting Information S2: Table S2.

For descriptive purposes, respondents were categorized into high‐ (score ≥ 8) and low‐satisfaction (score < 8) groups; this cut‐off was not prespecified. Because of the limited number of patient respondents, subgroup analyses of admission status, seclusion or restraint, and treatment‐component evaluations were interpreted descriptively. The only *p* value reported (Mann–Whitney *U* test) is nominal. Statistical analyses were performed using Python version 3.11 with NumPy 2.4 and SciPy 1.17.^25^

## RESULTS

### Respondent characteristics

Between May 2024 and March 2026, invitation flyers were handed to 61 patients aged 8–15 years and their families at discharge (Figure 1a). Responses were obtained from 78 participants, comprising 52 family respondents (85.2% of those invited) and 26 patient respondents (42.6%). The analytic sample comprised all 78 respondents. Ten patient responses were submitted within 3 min of a family response and were considered probable pairs from the same admission. Among family respondents, 47 (90.4%) were mothers and 5 (9.6%) were fathers; no grandparent responded.

**Figure 1:**
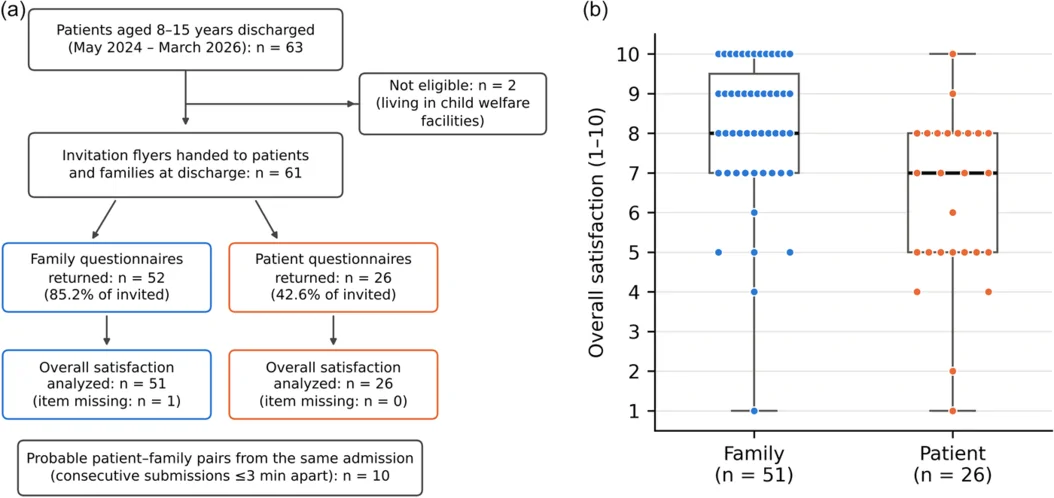
Participant flow and overall satisfaction scores by respondent type. (a) Participant flow. Of the 63 patients aged 8–15 years discharged between May 2024 and March 2026, two children living in child welfare facilities were not eligible; invitation flyers were handed at discharge to all 61 eligible patients and their families. Questionnaires were returned by 52 family members (85.2%) and 26 patients (42.6%); overall satisfaction was analyzed for 51 family and 26 patient respondents. Ten patient responses were submitted within 3 min of a family response (probable pairs). (b) Distribution of overall satisfaction scores (1–10). Each dot represents one respondent; boxes indicate interquartile ranges, horizontal lines medians, and whiskers ranges (family respondents, *n* = 51; patient respondents, *n* = 26).

Among patient respondents, the mean age at admission was 13.2 years (range, 8–15 years); 11 (42.3%) were male and 15 (57.7%) were female. Demographic and clinical characteristics are summarized in Table 1. Child‐level background items were not displayed to family respondents; therefore, family‐reported child characteristics are unavailable, and the clinical comparability of the two respondent groups could not be assessed. Seclusion or restraint during admission was reported by 26 of 52 family respondents (50.0%) and by 12 of 26 patient respondents (46.2%) (Table 1).

**Table 1:** Characteristics of respondents and patient‐reported characteristics.

Characteristic	Family respondents (*n* = 52)	Patient respondents (*n* = 26)
*Respondent*		
Mother	47 (90.4)	–
Father	5 (9.6)	–
Patient (self‐report)	–	26 (100)
*Seclusion or restraint during admission (reported by respondent)*		
Yes	26 (50.0)	12 (46.2)
No	26 (50.0)	13 (50.0)
Not answered	0 (0.0)	1 (3.8)
*Patient characteristics (patient‐reported)*		
Age at admission, years, mean (SD); median (range)	NA	13.2 (1.8); 14 (8–15)
Sex, male	NA	11 (42.3)
Sex, female	NA	15 (57.7)
Length of hospitalization		
<3 months	NA	0 (0.0)
3 to <6 months	NA	2 (7.7)
6 months to <1 year	NA	13 (50.0)
≥1 year	NA	11 (42.3)
Admission status at the time of admission		
Voluntary	NA	12 (46.2)
Medical protective	NA	13 (50.0)
Unknown	NA	1 (3.8)
Change in admission status during hospitalization		
Medical protective → voluntary (once)	NA	6 (23.1)
Voluntary → medical protective (once)	NA	1 (3.8)
Multiple changes	NA	4 (15.4)
Unknown	NA	11 (42.3)
Not answered	NA	4 (15.4)
Diagnosis reported by patient		
School non‐attendance or social withdrawal	NA	9 (34.6)
Depression or dysthymia	NA	5 (19.2)
Autism spectrum disorder	NA	4 (15.4)
Anxiety disorder	NA	3 (11.5)
Anorexia nervosa or eating disorder	NA	3 (11.5)
Other or unknown	NA	2 (7.7)
Reasons for admission (multiple answers)		
School non‐attendance	NA	16 (61.5)
Depressed mood	NA	12 (46.2)
Uncontrollable anxiety	NA	11 (42.3)
Recommendation by physician	NA	10 (38.5)
Self‐harm or suicidal thoughts	NA	7 (26.9)
Violent behavior at home	NA	4 (15.4)
Uncontrollable temper outbursts	NA	4 (15.4)
Inability to stop gaming	NA	4 (15.4)
Physical health problems	NA	4 (15.4)
Refusal or inability to eat	NA	4 (15.4)
Other	NA	3 (11.5)

*Note*: Values are *n* (%) unless otherwise indicated. Child‐level background items were included in the family questionnaire but, because of the branching settings of the online form, were not displayed to family respondents; no family‐reported child characteristics were therefore obtained. Seclusion or restraint was reported by both groups as one of the treatment components received. The item on change in admission status had no “did not change” option; “unknown” may therefore include patients whose status did not change. Diagnoses are those reported by patients in single choice or free text; one diagnosis per patient was coded (two patients who reported school non‐attendance together with autism spectrum disorder or dysthymia were coded as school non‐attendance).

Abbreviation: NA, not available.

### Admission‐related characteristics

Among patient respondents, admission status at the time of admission was voluntary for 12 (46.2%), medical protective for 13 (50.0%), and unknown for 1 (3.8%). Twelve patients (46.2%) reported that they had experienced seclusion or physical restraint, 13 (50.0%) reported that they had not, and 1 (3.8%) did not answer.

Length of hospitalization was 3 to <6 months for 2 patients (7.7%), 6 months to <1 year for 13 (50.0%), and ≥1 year for 11 (42.3%); no patient reported a stay of <3 months. Reasons for admission were collected using predefined multiple‐choice categories, including school non‐attendance, refusal or inability to eat, violent behavior at home, inability to stop gaming, uncontrollable temper outbursts, physical health problems, self‐harm or suicidal thoughts, depressed mood, uncontrollable anxiety, physician recommendation, and other. Diagnosis‐related items assessed whether the diagnosis had been explained or heard, and diagnoses were categorized as attention‐deficit/hyperactivity disorder, autism spectrum disorder, anorexia nervosa or eating disorder, depression, anxiety disorder, obsessive‐compulsive disorder, tic disorder, conduct disorder or oppositional defiant disorder, gaming disorder or gaming addiction, school refusal or social withdrawal, and other.

Changes in admission status were collected using four categories: one change from medical protective admission to voluntary admission, one change from voluntary admission to medical protective admission, multiple changes, and unknown. These items were obtained from patient respondents only. Primary diagnoses and reasons for admission are summarized in Table 1.

### Overall satisfaction

Overall satisfaction ratings were available for 51 family respondents (one did not answer) and all 26 patient respondents (Table 2; Figure 1b). The mean score was 8.14 (standard deviation [SD], 1.83; median, 8; interquartile range [IQR], 7–9.5; range, 1–10) among family respondents and 6.27 (SD, 2.15; median, 7; IQR, 5–8; range, 1–10) among patient respondents. The maximum score of 10 was given by 13 family respondents (25.5%) and 1 patient respondent (3.8%), and scores of 8 or higher by 36 family respondents (70.6%) and 10 patient respondents (38.5%).

**Table 2:** Distribution of overall satisfaction scores (1–10).

Score	Family respondents (*n* = 51)	Patient respondents (*n* = 26)
10 (very good)	13 (25.5)	1 (3.8)
9	12 (23.5)	1 (3.8)
8	11 (21.6)	8 (30.8)
7	9 (17.6)	4 (15.4)
6	1 (2.0)	1 (3.8)
5	3 (5.9)	7 (26.9)
4	1 (2.0)	2 (7.7)
3	0 (0.0)	0 (0.0)
2	0 (0.0)	1 (3.8)
1 (not good at all)	1 (2.0)	1 (3.8)
Score ≥ 8	36 (70.6)	10 (38.5)
Mean (SD)	8.14 (1.83)	6.27 (2.15)
Median (IQR)	8 (7–9.5)	7 (5–8)
Range	1–10	1–10
Family minus patient	Estimate (95% CI)	
Mean difference, all respondents	1.87 (0.87–2.86)	
Hodges–Lehmann median difference	2.0	
Sensitivity analyses for probable pairs (*n* = 10)		
Partially overlapping samples *t*‐test	1.87 (0.88–2.86)	
Excluding family members of probable pairs	1.78 (0.75–2.81)	*n* = 41 versus 26
Excluding patients of probable pairs	2.20 (0.81–3.59)	*n* = 51 versus 16
Within probable pairs, median difference	2.0 (family higher 8, equal 1, lower 1)	

*Note*: Values are *n* (%) unless otherwise indicated. One family respondent did not answer the item. IQR (linear interpolation). CI for the mean difference by Welch's method. Probable pairs: patient and family responses submitted within 3 min of each other, considered likely to concern the same admission. The partially overlapping samples *t*‐test (Tnew2) treats these as paired observations (correlation within pairs, *r* = 0.09). Supplementary information: Mann–Whitney *U* = 317, nominal *p* < 0.001; this test assumes independent samples.

Abbreviations: CI, confidence interval; IQR, interquartile range.

The mean difference (family minus patient respondents) was 1.87 points (95% CI, 0.87–2.86), and the Hodges–Lehmann median difference was 2 points. In sensitivity analyses addressing the 10 probable pairs, the 95% CI of the mean difference was 0.88–2.86 in the partially overlapping samples *t*‐test (within‐pair correlation, *r* = 0.09); the mean difference was 1.78 (95% CI, 0.75–2.81) after excluding the family responses and 2.20 (95% CI, 0.81–3.59) after excluding the patient responses of probable pairs. Assuming 17–26 pairs with within‐pair correlations from −0.5 to 0.5, the lower limit of the 95% CI ranged from 0.73 to 1.05. Within the probable pairs, the family member's score was higher in 8, equal in 1, and lower in 1 (median within‐pair difference, 2 points) (Table 2). For reference, the Mann–Whitney *U* test, which assumes independent samples, gave *U* = 317 (nominal *p* < 0.001; *r* = 0.43).

### Treatments received during hospitalization

Treatment components included interviews with the attending physician, psychologist, PSW, nursing staff, and occupational therapist; seclusion or restraint; group therapy or activities; pharmacotherapy; ward recreation; occupational therapy; cognitive behavioral therapy; in‐hospital classes; and discharge support meetings. For each item, respondents distinguished between not receiving the treatment component and not knowing whether it was received. Distributions are shown in Supporting Information S2: Table S2.

### Usefulness ratings of treatment components

Usefulness ratings were collected for interviews, pharmacotherapy, group therapy or activities, ward recreation, occupational therapy, cognitive behavioral therapy, in‐hospital classes, and seclusion or restraint using five response categories, ranging from not useful at all to very useful, with the additional option “had no opportunity to interact” (Supporting Information S2: Table S2).

### Professional involvement and professional evaluations

Professional involvement was assessed for physicians, nurses, psychologists, PSWs, occupational therapists, childcare workers, and teachers. Responses to the assignment item and the full ordinal distributions of usefulness ratings are shown for each professional group and respondent group in Table 3.

**Table 3:** Ratings of interactions with professional groups among respondents who rated the interaction.

Professional group/respondent	Assigned: yes/no/don't know/missing	No opportunity to interact	Item missing	Very useful	Useful	Neither	Not very useful	Not useful at all	Useful or very useful, *n*/*N* (%)
Child and adolescent psychiatrists									
Family	47/4/0/1	0	0	37	14	0	1	0	51/52 (98.1)
Patient	24/0/2/0	0	0	11	7	5	3	0	18/26 (69.2)
Nurses									
Family	42/4/3/3	0	0	29	19	2	1	1	48/52 (92.3)
Patient	24/0/2/0	0	0	11	8	5	2	0	19/26 (73.1)
Clinical psychologists									
Family	22/6/21/3	9	0	15	18	8	2	0	33/43 (76.7)
Patient	11/7/8/0	6	0	6	11	2	1	0	17/20 (85.0)
PSWs									
Family	28/7/13/4	12	2	11	14	11	1	1	25/38 (65.8)
Patient	18/4/4/0	4	0	9	10	2	1	0	19/22 (86.4)
Teachers									
Family	35/8/4/5	7	1	27	11	4	1	1	38/44 (86.4)
Patient	15/2/9/0	2	0	13	7	2	0	2	20/24 (83.3)

*Note*: Family respondents, *n* = 52; patient respondents, *n* = 26. The denominator (*N*) comprises respondents who rated the interaction; respondents who selected “had no opportunity to interact” or left the item blank were excluded. The same rule was applied to all professional groups and both respondent groups. “Assigned” is a separate item asking whether the professional had been assigned to the patient (assigned, not assigned, or don't know); it is shown for description and was not used to define denominators. Child and adolescent psychiatrists correspond to the questionnaire row “attending physician” and nurses to the row “nurses” (Supporting Information S1). For family respondents rating PSWs, 12 reported no opportunity to interact and 2 left the item blank (*N* = 52 − 12 − 2 = 38). Comparisons between family and patient respondents are descriptive. Confidence intervals and tests are not reported because a patient and a family member could both have responded about the same admission, and the anonymous responses could not be linked.

Abbreviation: PSW, psychiatric social worker.

Denominators comprised respondents who rated their interaction with each professional group; respondents who indicated no opportunity to interact or left the item blank were excluded, using the same rule for all groups (Table 3).

Child and adolescent psychiatrists were rated as useful or very useful by 51 of 52 family respondents (98.1%) and 18 of 26 patient respondents (69.2%).

Nurses were rated as useful or very useful by 48 of 52 family respondents (92.3%) and 19 of 26 patient respondents (73.1%).

Clinical psychologists were rated as useful or very useful by 33 of 43 family respondents (76.7%) and 17 of 20 patient respondents (85.0%); 9 family and 6 patient respondents reported no opportunity to interact.

PSWs were rated as useful or very useful by 25 of 38 family respondents (65.8%) and 19 of 22 patient respondents (86.4%). Twelve family respondents and four patient respondents reported no opportunity to interact with a PSW, and two family respondents did not answer the item.

Teachers were rated as useful or very useful by 38 of 44 family respondents (86.4%) and 20 of 24 patient respondents (83.3%); 7 family and 2 patient respondents reported no opportunity to interact, and one family respondent did not answer the item (Table 3; Figure 2). These comparisons are descriptive; no CIs or tests were calculated because patient and family responses could not be assumed to be independent.

**Figure 2:**
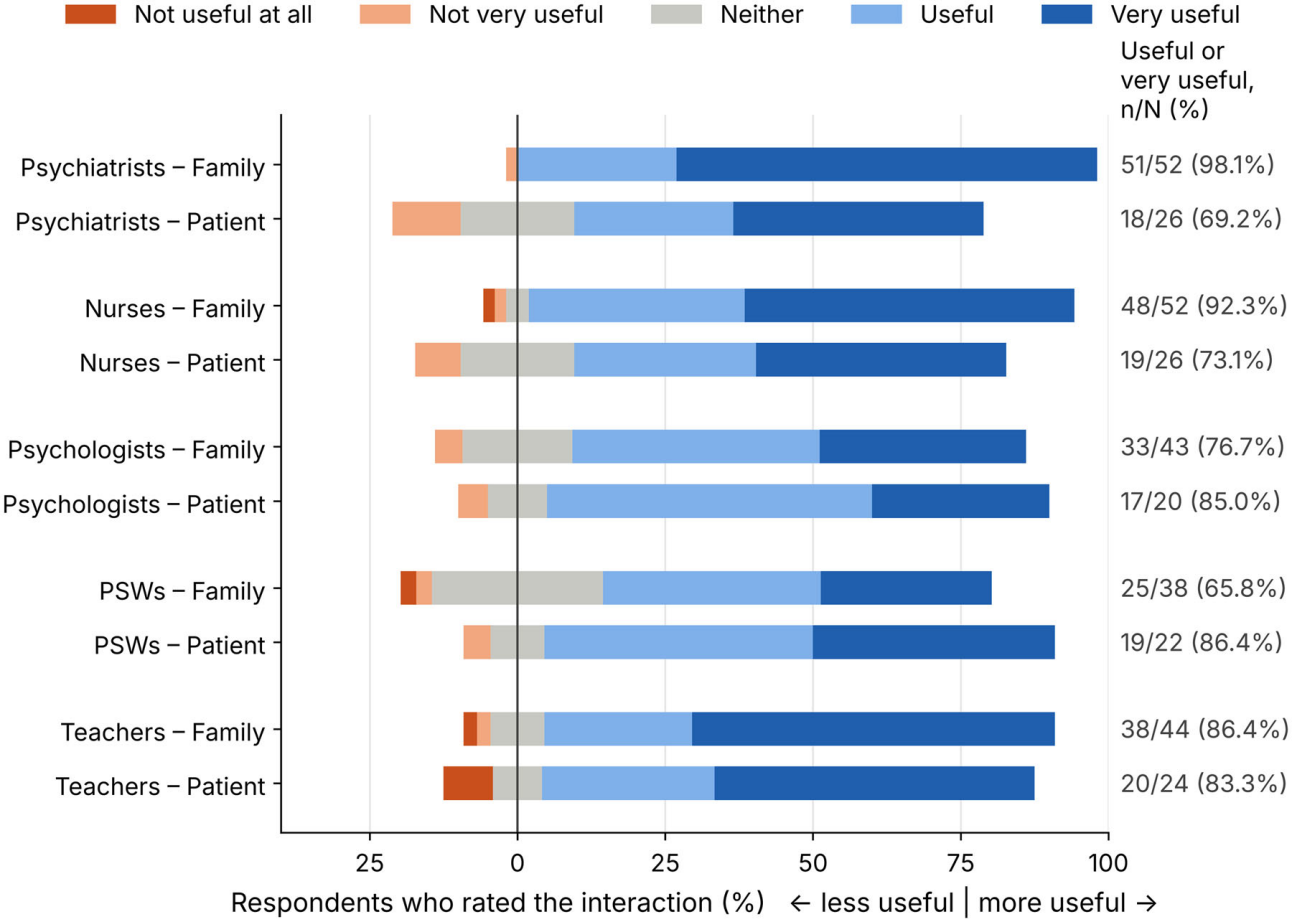
Usefulness ratings of interactions with professional groups by respondent type. Diverging stacked bars show the distribution of five‐point usefulness ratings for child and adolescent psychiatrists, nurses, clinical psychologists, psychiatric social workers (PSWs), and teachers among respondents who rated their interaction with each group (respondents indicating no opportunity to interact or leaving the item blank were excluded). Ratings of “useful” and “very useful” are plotted to the right of zero, and “neither” is centered on zero. Numbers at right give the count and percentage rating the group as useful or very useful (*n*/*N*). Denominators and full distributions are reported in Table 3. Comparisons are descriptive; no confidence intervals or tests are shown because patient and family responses may not be independent.

### Satisfaction groups

Classification by satisfaction level was possible for 77 respondents: 46 had scores of ≥8 and 31 had scores of <8.

Among respondents with scores of ≥8, 36 (78.3%) were family respondents and 10 (21.7%) were patient respondents. Among those with scores of <8, 15 (48.4%) were family respondents and 16 (51.6%) were patient respondents.

Among family respondents, positive evaluations of child and adolescent psychiatrists were reported by all 36 respondents (100%) in the high‐satisfaction group and 14 of 15 (93.3%) in the low‐satisfaction group. Positive evaluations of nurses were reported by 35 of 36 respondents (97.2%) and 12 of 15 respondents (80.0%), respectively (Table 4).

**Table 4:** Respondents by overall satisfaction level.

Characteristic	Score ≥ 8 (*n* = 46)	Score < 8 (*n* = 31)
Family respondents	36 (78.3)	15 (48.4)
Patient respondents	10 (21.7)	16 (51.6)
Family respondents only		
Psychiatrists rated useful or very useful	36/36 (100.0)	14/15 (93.3)
Nurses rated useful or very useful	35/36 (97.2)	12/15 (80.0)

*Note*: Values are *n* (%) or *n*/*N* (%). Seventy‐seven respondents with an overall satisfaction score were classified (one family respondent did not answer). The cut‐off of 8 was chosen descriptively and was not prespecified.

### Admission status, behavioral restriction, and satisfaction in patient respondents

Among the 25 patients with known admission status, the mean satisfaction score was 6.8 (median, 7.5; *n* = 12) for voluntary admission and 5.8 (median, 7; *n* = 13) for medical protective admission.

Among the 25 patients who answered the seclusion or restraint item, the mean satisfaction score was 6.6 (median, 7; *n* = 12) for those who reported seclusion or physical restraint and 6.4 (median, 7; *n* = 13) for those who did not.

Admission status was unknown for one patient and the seclusion or restraint item was unanswered by another; these single observations were not summarized (Table 5). Because of the limited subgroup sample sizes, formal inferential analyses were not performed, and these findings are presented descriptively.

**Table 5:** Overall satisfaction by admission status and seclusion or restraint (patient respondents, *n* = 26).

Patient‐reported subgroup	*n*	Mean (median) satisfaction
Admission status at the time of admission		
Voluntary	12	6.8 (7.5)
Medical protective	13	5.8 (7)
Unknown	1	Not summarized
Seclusion or restraint during admission		
Yes	12	6.6 (7)
No	13	6.4 (7)
Not answered	1	Not summarized

*Note*: Descriptive only; no inferential tests were performed because of small subgroup sizes. Categories with one respondent are not summarized.

## DISCUSSION

### Main findings

This exploratory cross‐sectional study found differences between child and adolescent patients and family respondents in evaluations of psychiatric inpatient treatment. Family members reported higher overall satisfaction than patients, with a mean difference of nearly two points on a 10‐point scale and a 95% CI that excluded zero in all sensitivity analyses addressing dependence between the two groups. A higher proportion of family members than patients also rated child and adolescent psychiatrists and nurses as useful, whereas proportions for clinical psychologists, PSWs, and teachers were similar or higher among patients; these profession‐specific comparisons were descriptive. These findings suggest that patients and families evaluate inpatient care from different perspectives.

Lower patient satisfaction may reflect a weaker therapeutic alliance or less perceived respect, privacy, autonomy, or involvement in care, although none of these was measured here. Because satisfaction explains only limited variance in later clinical outcomes,^10^ the difference is best regarded as a difference in experience of care that warrants further study.

The directionality of these findings is consistent with previous research showing that parents generally report higher satisfaction than adolescents.^9^, ^20^ Previous matched‐dyad studies reported little to moderate agreement between adolescents and their parents^9^, ^20^, ^26^, ^27^; the present unlinked comparison adds descriptive information on how evaluations were distributed within a Japanese inpatient unit and cannot estimate disagreement within individual patient–family dyads. Furthermore, as previous studies suggest,^20^, ^28^ specific professional ratings may partly measure a general satisfaction factor or overall impression of care rather than entirely distinct constructs.

### Patient satisfaction as an experience‐of‐care indicator

An important implication of this study is that patient‐reported satisfaction may serve as an experience‐of‐care indicator for quality improvement in child and adolescent psychiatric inpatient care.^5^, ^6^, ^7^, ^16^, ^28^ Rather than representing a treatment outcome in itself, satisfaction may reflect therapeutic relationships, communication, perceived respect, and the inpatient environment during hospitalization. Previous studies have reported associations between young people's satisfaction and later clinical outcomes, although these associations were modest.^9^, ^10^ For children and adolescents, who are developing autonomy and self‐regulation, feeling respected and understood may be particularly important. If treatment explanations are insufficient or privacy and therapeutic relationships are perceived negatively, patients may be less likely to accept treatment recommendations after discharge.^8^, ^9^

Family satisfaction may reflect different dimensions of care, including safety, symptom stabilization, communication with professionals, and relief of family burden.^11^, ^12^ Although important, these factors may not fully capture patients' readiness to continue treatment. Therefore, high family satisfaction does not guarantee that patients experienced care in the same way, and assessing patient‐reported satisfaction alongside family reports may be useful for quality improvement and discharge planning. Satisfaction is therefore best interpreted as an experience‐of‐care measure rather than as a proxy for clinical quality or treatment outcome.

### Differences between patient and family perspectives

Differences between patient and family satisfaction may reflect differences in roles, expectations, and experiences during hospitalization.^14^, ^15^ Families primarily interact with the treatment team through scheduled meetings, explanations, and discharge planning, and may base their evaluations on professionalism, clarity of explanations, and reassurance regarding patient safety. Consistent with this, family respondents rated child and adolescent psychiatrists and nurses positively.

Patients experience hospitalization directly through daily routines, ward rules, restrictions, peer relationships, privacy, autonomy, and informal interactions with staff. Hospitalization may involve loss of control, separation from familiar environments, and interpersonal stress. Consequently, satisfaction may decrease if patients do not feel sufficiently heard, respected, or involved in decisions.

Among respondents who reported interaction with PSWs, the proportion rating them as useful was higher among patients than among family respondents (86.4% vs. 65.8%); this descriptive comparison was based on small numbers and should not be read as evidence of a difference.

PSWs in our setting typically focus on environmental adjustments, school reintegration, and discharge planning rather than direct symptom management or enforcing ward rules. Patients may therefore have perceived PSWs as advocates for their future rather than as authority figures; however, this was not measured, and the numbers were small.

### Clinical implications

These findings have several implications for psychiatric practice. First, satisfaction should be evaluated independently in child and adolescent patients and their families, rather than relying on parental reports as proxies for patients' experiences. High family satisfaction does not necessarily indicate that patients perceived inpatient care as acceptable, respectful, or therapeutically meaningful.

Second, lower patient satisfaction should prompt reflection on communication, shared decision‐making, perceived coercion, privacy, and explanation of treatment goals, all of which are potentially modifiable. Providing structured opportunities for patients to express concerns, participate in treatment planning, and review their experiences may improve therapeutic engagement.

Third, multidisciplinary care should also be evaluated from the patient's perspective. Although psychiatrists and nurses are central to inpatient care, patients may perceive them less positively than families do. Improving daily communication, explaining treatment decisions and restrictions, and validating patients' perspectives are potential targets for improvement. PSWs may further contribute by facilitating continuity of care, school reintegration, and family adjustment after discharge.

Finally, patient satisfaction may be useful in discharge planning. In this study, satisfaction was assessed after discharge. If it were assessed before discharge, patients with low satisfaction could be offered additional engagement, clearer explanations of the treatment plan, or enhanced transition support, and such assessment may help identify children and adolescents requiring additional support during transition from inpatient to outpatient or community care. Behavioral restrictions require careful interpretation. In the exploratory patient‐only analysis, mean satisfaction was not lower among patients who experienced seclusion or physical restraint compared with those who did not (6.6 vs. 6.4). This finding should not be interpreted as evidence that behavioral restrictions improve satisfaction because it was based on a small subgroup and is susceptible to sparse‐data instability, reverse causation, and confounding by indication. Patients requiring seclusion or restraint may have had greater baseline clinical severity while also receiving more intensive multidisciplinary intervention and crisis stabilization, which could influence retrospective evaluations of safety, care, or therapeutic relationships. Future longitudinal studies incorporating baseline severity measures and matched patient‐family data are needed to examine the association between behavioral restrictions and satisfaction. From a trauma‐informed care perspective,^29^ these findings should not be interpreted as justification for coercive interventions. Although behavioral restrictions are permitted under the Mental Health and Welfare Act only to manage imminent clinical risks and maintain ward safety, they represent significant intrusions on young patients' autonomy and dignity.^22^, ^30^, ^31^

### Limitations

Several limitations should be acknowledged. First, the cross‐sectional design precluded assessment of causal relationships between satisfaction and clinical outcomes. Second, the relatively small sample size, particularly for patient respondents, limited statistical power and generalizability; therefore, analyses of admission status and behavioral restrictions should be interpreted cautiously, and the imprecise subgroup estimates should not be interpreted as evidence of no clinically meaningful association. Third, responses were unlinked. Although 10 probable patient–family pairs were identified from submission times and sensitivity analyses gave similar results, other pairs may have been missed, and the unlinked comparison cannot estimate disagreement between individual patients and their own parents. Because child characteristics were not obtained from family respondents, the clinical comparability of the two groups could not be assessed, and the observed difference may partly reflect differences in clinical background and in which patients and families responded. For the same reason, the profession‐specific comparisons were reported descriptively, without CIs or tests. Furthermore, selection bias is possible because more engaged or more satisfied family members may have been more likely to complete the survey, potentially inflating family satisfaction scores; the lower response rate among patients (42.6%) than among families (85.2%) also means that nonresponse bias cannot be excluded. Children living in child welfare facilities were not eligible, so the findings do not represent their experiences of inpatient care. The ceiling effect among family respondents (25.5% scoring 10) limits discrimination at the upper end of the scale. Fourth, family respondents were predominantly mothers, limiting the generalizability to fathers or other guardians. Although this respondent imbalance may reflect caregiving roles in Japanese child and adolescent psychiatric settings, paternal perspectives on inpatient care and discharge readiness remain underrepresented. Fifth, denominators varied across analyses because of item‐level missing data and differences in whether respondents had interacted with specific professional groups. Finally, satisfaction ratings may have been influenced by illness severity, treatment context, admission status, behavioral restrictions, and survey timing (the interval between discharge and response varied), which could not be fully controlled. Overall satisfaction was also assessed using an unstandardized, single‐item 10‐point scale. Although such measures are widely used in routine clinical practice and reduce respondent burden, they provide less psychometric evaluation of construct validity, internal consistency, and test‐retest reliability than validated multidimensional instruments.^32^ In addition, the online form contained errors that were not detected before data collection: in the patient form, the cognitive behavioral therapy row of the treatment‐received item was labeled “occupational therapy,” and in both forms seclusion or restraint was listed twice. Patient responses to the mislabeled row were not analyzed.

Despite these limitations, the present study provides exploratory descriptive evidence on how child and adolescent patients and their families evaluate inpatient care. Because no pre‐specified progression criteria were defined, we do not claim that this survey protocol is feasible.^33^ The findings highlight the need for larger multicenter collaborative studies using matched patient–family dyads to determine the generalizability of these results and clarify their associations with post‐discharge outcomes, treatment adherence, and continuity of care.

## CONCLUSIONS

In this exploratory survey, child and adolescent patients reported lower overall satisfaction with psychiatric inpatient treatment than family respondents. Whether lower patient satisfaction reflects therapeutic alliance, perceived respect, privacy, or autonomy, which were not measured here, remains to be examined in matched‐dyad research. Although family satisfaction is important, it cannot replace patients' own evaluations. Routine assessment of both patient and family perspectives may help identify unmet needs and support quality improvement. These findings may inform the design of future multicenter studies evaluating patient‐ and family‐reported satisfaction.

## AUTHOR CONTRIBUTIONS

**Keita Yamamoto**: Investigation; data curation; writing—original draft. **Masahide Usami**: Conceptualization; methodology; formal analysis; writing—original draft; writing—review and editing; supervision; project administration; funding acquisition. Both authors contributed substantially to the study, providing feedback on the interpretation of the results and manuscript preparation. Both authors have read and approved the final version of the manuscript.

## CONFLICT OF INTEREST STATEMENT

The authors declare no conflicts of interest.

## ETHICS APPROVAL STATEMENT

This study was approved by the Ethics Committee of the National Center for Global Health and Medicine (now the Japan Institute for Health Security; approval no. 004842) and was conducted in accordance with the Declaration of Helsinki.

## PATIENT CONSENT STATEMENT

The requirement for written informed consent was waived because this study used an anonymous web‐based questionnaire. Child and adolescent participants and family respondents reviewed the study information statement before completing the questionnaire, and selecting “I agree” in the consent item before proceeding to the questionnaire was regarded as consent to participate, in accordance with the procedures approved by the ethics committee.

## CLINICAL TRIAL REGISTRATION

Not applicable.

## Supporting information

Supporting File 1.

Supporting File 2.

## Data Availability

The data that support the findings of this study are not publicly available because of institutional data sharing policies and restrictions related to participant privacy and ethical approval. Data may be available from the corresponding author upon reasonable request and subject to approval by the relevant institutional review board and the Japan Institute for Health Security.
