# Supplementary material for: Patient‐ and family‐reported satisfaction in child and adolescent psychiatric inpatient care: An exploratory descriptive survey
Source: PCN Rep. 2026 Nov 15;5(4):e70445. doi: 10.1002/pcn5.70445 (PMC13770296; doi:10.1002/pcn5.70445)
Supplement: Supplementary file 1 [file PCN5-5-e70445-s001.docx]

**Supplementary File S1. Questionnaire as displayed to respondents, English translation, and coding**

Manuscript: Patient- and Family-Reported Satisfaction in Child and Adolescent Psychiatric Inpatient Care: An Exploratory Descriptive Survey (PCNR-2026-0177, revision R3)

Part A gives an item-by-item English translation in the order and format displayed; Part B explains how each item was coded for analysis; Part C reproduces the Japanese form as screen captures (Microsoft Forms print view, 15 pages).

**Part A. Items and response options as displayed (English translation)**

Item numbers are those shown in Microsoft Forms. Japanese wording is given in parentheses for key terms. Furigana (phonetic readings) printed in the patient form are omitted from the translation. Single choice: one option only. Multiple choice: any number of options. Grid: one option per row.

| **Item** | **Question (English translation)** | **Response format and options as displayed** |
| --- | --- | --- |
| 1 | Information statement and consent (all respondents) | Single choice: I agree (同意する) / I do not agree (同意しない) |
| 2 | Hospital name (optional) | Single choice: Kohnodai Hospital; Okayama Psychiatric Medical Center; Tokyo Metropolitan Children’s Medical Center; Nagano Prefectural Mental Wellness Center Komagane; Other |
| 3 | Who is responding? (回答される方) | Single choice: The patient (the child who was admitted) (本人); Family (mother); Family (father); Family (grandparent). This item determined which branch was displayed. |
|  | Patient branch: Admission information (for the patient) |  |
| 4 | Your sex | Single choice: Boy; Girl |
| 5 | Your age at admission | Number |
| 6 | Length of your hospitalization | Single choice: <3 months; 3 to <6 months; 6 months to <1 year; ≥1 year |
| 7 | Reasons for admission | Multiple choice: School non-attendance; Refusing to eat or unable to eat; Violent behavior at home; Unable to stop gaming; Temper outbursts that would not stop; Poor physical condition; Self-harm or wanting to die; Feeling depressed; Anxiety that would not stop; Recommended by the attending physician; Other |
| 8 | Have you been told your diagnosis? | Single choice: Yes; I was told but forgot; I have never been told; Other (free text) |
| 9 | Please tell us the diagnosis for which you were admitted and treated | Single choice: ADHD; Autism spectrum disorder; Anorexia; Depression; Anxiety disorder; Obsessive-compulsive disorder; Tic disorder; Conduct disorder/oppositional defiant disorder; Gaming disorder/gaming addiction; School non-attendance/social withdrawal; Other (free text) |
| 10 | At admission, was your admission medical protective or voluntary? | Single choice: Medical protective admission; Voluntary admission; Don’t know |
| 11 | Did your admission status change during hospitalization? | Single choice: Changed once from medical protective to voluntary; Changed once from voluntary to medical protective; Changed several times; Don’t know (no “did not change” option was offered) |
|  | Family branch: Admission information (for guardians). Because of the branching settings, items 12–19 were not displayed to family respondents, and no family respondent answered them. |  |
| 12–19 | Child’s sex; age; length of hospitalization; reasons for admission; whether the diagnosis had been explained; diagnosis; admission status at admission; admission status at discharge | Options corresponding to items 4–11 |
|  | Patient section: About your hospital life (for the patient) |  |
| 20 | Please tell us which professionals were assigned to you (自分の担当だった職種) | Grid; rows: Physician; Nurse; Psychologist; Social worker; Occupational therapist; Childcare worker; Teacher (homeroom). Columns: Not assigned (担当でなかった); Don’t know (わからない); Assigned (担当だった) |
| 21 | Please tell us what you thought of your inpatient treatment | Rating 1–10; anchors: 1 = not good at all (全く良くなかった); 10 = very good (すごく良かった) |
| 22 | Please tell us which treatments you received during your hospital life, part 1 | Grid; rows: Interview (attending physician); Seclusion/restraint; Interview (psychologist); Interview (social worker); Interview (nursing); Interview (occupational therapist). Columns: Did not receive; Don’t know; Received |
| 23 | Please tell us which treatments you received during your hospital life, part 2 | Grid; rows as displayed: Group therapy/group activities; Pharmacotherapy; Ward recreation; Occupational therapy; Occupational therapy [label error: this row was intended to be cognitive behavioral therapy]; In-hospital class; Seclusion/restraint; Discharge support meeting (medical protective admission only). Columns: Did not receive; Don’t know; Received |
| 24 | Please tell us which treatments you found useful during your hospital life | Grid; rows: Interviews; Pharmacotherapy; Group therapy/group activities; Ward recreation, etc.; Occupational therapy; Cognitive behavioral therapy; In-hospital class; Seclusion/restraint. Columns: Not useful at all; Not very useful; Neither; Useful; Very useful; Had no opportunity to interact (交流する機会がなかった) |
| 25 | Please tell us which of the staff in charge of you (担当者) you found useful during your hospital life | Grid; rows: Relationship with the attending physician (主治医); with nurses (看護師); with psychologists; with social workers; with occupational therapists; with childcare workers; with teachers. Columns as in item 24, including “had no opportunity to interact” |
|  | Family section: Your child’s treatment (for guardians) |  |
| 26 | Please tell us which professionals were assigned to your child | Grid as in item 20 |
| 27 | Please tell us about the inpatient treatment | Rating 1–10; anchors as in item 21 |
| 28 | Please tell us which treatments your child received, part 1 | Grid as in item 22. Instruction: “Interviews” means regular interviews (e.g., once a week or once a month); please also include the discharge support meeting for medical protective admission. |
| 29 | Please tell us which treatments your child received, part 2 | Grid; rows: Group therapy; Pharmacotherapy; Ward recreation; Occupational therapy; Cognitive behavioral therapy; Use of in-hospital class; Seclusion/restraint; Discharge support meeting (medical protective admission only). Columns as in item 22 |
| 30 | Please tell us which treatments you found useful for your child’s inpatient treatment | Grid as in item 24 |
| 31 | Please tell us which of the staff in charge (担当者) you found useful for your child’s inpatient treatment | Grid as in item 25 |

**Part B. Coding used in the analyses**

| **Variable (item)** | **Coding** |
| --- | --- |
| Respondent type (item 3) | Patient vs. family respondent (mother, father, grandparent). No grandparent responded. |
| Overall satisfaction (items 21, 27) | Score 1–10 as selected; blank = missing (one family respondent). |
| Assignment of professionals (items 20, 26) | Assigned / not assigned / don’t know; blank = missing. This item asked about assignment, not contact. Reported descriptively in Table 3 and not used to define denominators. |
| Treatments received (items 22–23, 28–29) | Each row coded as received / not received / don’t know; blank = not answered. This was a grid with one choice per row, not a multiple-selection item, so “not received” and “don’t know” were explicit responses (Supplementary Table S2a). |
| Seclusion/restraint (items 22–23, 28–29) | Listed in both grids. The first appearance (items 22, 28) was analyzed; counts for the second appearance are given in the note to Supplementary Table S2. |
| Cognitive behavioral therapy received (items 23, 29) | Patient form: the row intended for cognitive behavioral therapy was displayed as “occupational therapy,” so respondents saw the incorrect label; this row was not analyzed. Family form: correct label; analyzed. |
| Usefulness of treatments (items 24, 30) | Five categories as displayed. “Had no opportunity to interact” is shown separately as “no opportunity”; blank = not answered. |
| Usefulness of staff (items 25, 31) | Rows referred to the staff in charge of the patient (担当者). Five categories; “useful” or “very useful” = positive evaluation. Respondents who selected “had no opportunity to interact” or left the row blank were excluded from the denominator. Rows analyzed: attending physician (reported as child and adolescent psychiatrists, because the attending physicians in this unit were child and adolescent psychiatrists), nurses, psychologists (reported as clinical psychologists), social workers (reported as psychiatric social workers, PSWs), and teachers. Occupational therapists and childcare workers were not analyzed because few respondents rated them. |
| Reasons for admission (item 7) | Each option coded selected / not selected. |
| Change in admission status (item 11) | As selected; because no “did not change” option was offered, “don’t know” may include patients whose status did not change. |
| Diagnosis (item 9) | Single choice or free text; one diagnosis per patient, coded as described in the note to Table 1. |
| Branching error (one patient response) | One respondent who selected “the patient” in item 3 answered items 4–11 and then items 12–31 of the family branch. The responses to items 26–31 were analyzed as this patient’s responses, so that all 26 patient respondents are included. |

**Part C. Screen captures of the questionnaire (Microsoft Forms print view, in Japanese)**

Pages 1–15 of the form. Page 1: title; page 2: information statement and consent (item 1); page 3: items 2–3; pages 4–6: patient admission information (items 4–11); pages 7–9: family admission information (items 12–19); pages 10–12: patient section (items 20–25); pages 13–15: family section (items 26–31). The captures were printed from the live Microsoft Forms questionnaire on August 27, 2026; the form was not modified after data collection began. The browser print header and footer have been removed.
