# Supplementary material for: Patient‐ and family‐reported satisfaction in child and adolescent psychiatric inpatient care: An exploratory descriptive survey
Source: PCN Rep. 2026 Nov 15;5(4):e70445. doi: 10.1002/pcn5.70445 (PMC13770296; doi:10.1002/pcn5.70445)
Supplement: Supplementary file 2 [file PCN5-5-e70445-s002.docx]

**Supplementary Table S2. Treatment components received and usefulness ratings, by respondent group**

(a) Treatment components received during hospitalization, n (%)

| **Treatment component** | **Group** | **Received** | **Not received** | **Don't know** | **Not answered** |
| --- | --- | --- | --- | --- | --- |
| Interview with attending physician | Family | 51 (98.1) | 1 (1.9) | 0 (0.0) | 0 (0.0) |
|  | Patient | 26 (100.0) | 0 (0.0) | 0 (0.0) | 0 (0.0) |
| Seclusion or restraint | Family | 26 (50.0) | 26 (50.0) | 0 (0.0) | 0 (0.0) |
|  | Patient | 12 (46.2) | 13 (50.0) | 0 (0.0) | 1 (3.8) |
| Interview with psychologist | Family | 31 (59.6) | 7 (13.5) | 14 (26.9) | 0 (0.0) |
|  | Patient | 13 (50.0) | 6 (23.1) | 6 (23.1) | 1 (3.8) |
| Interview with PSW | Family | 29 (55.8) | 8 (15.4) | 14 (26.9) | 1 (1.9) |
|  | Patient | 16 (61.5) | 6 (23.1) | 4 (15.4) | 0 (0.0) |
| Interview with nursing staff | Family | 28 (53.8) | 11 (21.2) | 12 (23.1) | 1 (1.9) |
|  | Patient | 14 (53.8) | 2 (7.7) | 10 (38.5) | 0 (0.0) |
| Interview with occupational therapist | Family | 0 (0.0) | 24 (46.2) | 26 (50.0) | 2 (3.8) |
|  | Patient | 0 (0.0) | 22 (84.6) | 4 (15.4) | 0 (0.0) |
| Group therapy/activities | Family | 43 (82.7) | 3 (5.8) | 5 (9.6) | 1 (1.9) |
|  | Patient | 24 (92.3) | 0 (0.0) | 2 (7.7) | 0 (0.0) |
| Pharmacotherapy | Family | 31 (59.6) | 18 (34.6) | 2 (3.8) | 1 (1.9) |
|  | Patient | 12 (46.2) | 12 (46.2) | 2 (7.7) | 0 (0.0) |
| Ward recreation | Family | 48 (92.3) | 2 (3.8) | 1 (1.9) | 1 (1.9) |
|  | Patient | 24 (92.3) | 0 (0.0) | 2 (7.7) | 0 (0.0) |
| Occupational therapy | Family | 15 (28.8) | 9 (17.3) | 27 (51.9) | 1 (1.9) |
|  | Patient | 8 (30.8) | 5 (19.2) | 13 (50.0) | 0 (0.0) |
| Cognitive behavioral therapy | Family | 12 (23.1) | 12 (23.1) | 26 (50.0) | 2 (3.8) |
|  | Patient | Not assessed (label error; see note) |  |  |  |
| In-hospital classes | Family | 46 (88.5) | 6 (11.5) | 0 (0.0) | 0 (0.0) |
|  | Patient | 25 (96.2) | 1 (3.8) | 0 (0.0) | 0 (0.0) |
| Discharge support meeting | Family | 25 (48.1) | 18 (34.6) | 5 (9.6) | 4 (7.7) |
|  | Patient | 16 (61.5) | 9 (34.6) | 1 (3.8) | 0 (0.0) |

Family respondents, n = 52; patient respondents, n = 26. Each component was a row of a grid with one choice per row (received, did not receive, don’t know; Supplementary File S1, items 22–23 and 28–29); “not answered” indicates a blank row. All components were reported by respondents and were not verified against medical records. Seclusion or restraint appeared in both grids; the first appearance is shown (the second gave family 25 received, 25 not received, 1 don’t know, 1 not answered; patients 12 received, 14 not received). In the patient form, the row intended for cognitive behavioral therapy was displayed as “occupational therapy” (occupational therapy appeared twice), so patients saw the incorrect label; patient responses to this row are not reported. The family form displayed the correct label. The discharge support meeting row was labeled “medical protective admission only” in both forms, so “not received” may include patients who were admitted voluntarily.

(b) Usefulness ratings of treatment components, n

| **Treatment component** | **Group** | **Very useful** | **Useful** | **Neither** | **Not very useful** | **Not useful at all** | **No opportunity** | **Not answered** |
| --- | --- | --- | --- | --- | --- | --- | --- | --- |
| Interviews | Family (n = 52) | 32 | 17 | 1 | 0 | 1 | 1 | 0 |
|  | Patient (n = 26) | 8 | 10 | 6 | 1 | 1 | 0 | 0 |
| Pharmacotherapy | Family (n = 52) | 13 | 9 | 12 | 2 | 2 | 13 | 1 |
|  | Patient (n = 26) | 2 | 5 | 8 | 2 | 1 | 6 | 2 |
| Group therapy/activities | Family (n = 52) | 22 | 23 | 1 | 0 | 0 | 5 | 1 |
|  | Patient (n = 26) | 10 | 7 | 4 | 0 | 1 | 3 | 1 |
| Ward recreation | Family (n = 52) | 28 | 20 | 1 | 0 | 0 | 3 | 0 |
|  | Patient (n = 26) | 12 | 5 | 2 | 5 | 1 | 1 | 0 |
| Occupational therapy | Family (n = 52) | 4 | 12 | 16 | 0 | 2 | 17 | 1 |
|  | Patient (n = 26) | 1 | 5 | 11 | 0 | 0 | 7 | 2 |
| Cognitive behavioral therapy | Family (n = 52) | 7 | 11 | 12 | 0 | 1 | 20 | 1 |
|  | Patient (n = 26) | 4 | 2 | 8 | 0 | 0 | 9 | 3 |
| In-hospital classes | Family (n = 52) | 24 | 15 | 3 | 3 | 0 | 7 | 0 |
|  | Patient (n = 26) | 12 | 10 | 1 | 0 | 2 | 1 | 0 |
| Seclusion or restraint | Family (n = 52) | 5 | 13 | 7 | 2 | 1 | 21 | 3 |
|  | Patient (n = 26) | 1 | 0 | 7 | 4 | 4 | 8 | 2 |

“No opportunity” indicates that the respondent selected “had no opportunity to interact,” which was displayed as a sixth option in every row (Supplementary File S1, items 24 and 30). The cognitive behavioral therapy row was correctly labeled in this item in both forms. Items (a) and (b) were answered separately; therefore, the number of respondents who rated seclusion or restraint in (b) can exceed the number who reported receiving it in (a). Ratings are descriptive; no between-group tests were performed. PSW, psychiatric social worker.
